# Changes in the smoking behavior of pregnant women andtheir family members during pregnancy: A cross-sectional study in China

**DOI:** 10.18332/tid/85493

**Published:** 2018-04-10

**Authors:** Tianyu Tan, Lili Shi, Xiaowen Chen, Yuyang Cai

**Affiliations:** 1 School of Public Health, Shanghai Jiao Tong University, School of Medicine, Shanghai, China; 2 Xinhua Hospital, Shanghai Jiao Tong University, School of Medicine, Shanghai, China; 3 Children’s Hospital of Fudan University, Shanghai, China

**Keywords:** China, smoking behavior changes, pregnancy, environmental tobacco smoke

## Abstract

**INTRODUCTION:**

Studies regarding the changes in smoking behavior of pregnant women have been mostly conducted in high income countries but rarely in China. This study thus focused on investigating the changes in smoking behavior among pregnant women and their family members in China, both before and during pregnancy.

**METHODS:**

A cross-sectional study was carried out at nine Women and Children’s Hospitals in Shanghai, China, in 2014. A total of 2831 gestational households were recruited. The chi-squared test, paired sample t-test and logistical regression analysis were used during statistical analysis.

**RESULTS:**

The prevalence of smoking for all household members significantly declined during pregnancy: 76.2% of the pregnant women, 19.2% of their husbands and 14.0% of other family members quit smoking entirely. The average daily cigarette consumption rate decreased from 5.9 to 0.9 among pregnant women, 11.4 to 9.5 for husbands, and 11.4 to 9.5 for other family members (paired sample t-test, p<0.001). The likelihood that husbands continued smoking during pregnancy was significantly lower for those with a shorter history of smoking, had a lower daily cigarette consumption rate and a household registration in Shanghai.

**CONCLUSIONS:**

The prevalence of smoking among pregnant women, husbands and other family members significantly declines during pregnancy. Thus, pregnancy is most likely a key period in which to provide families with health education regarding the effects of smoking, both during the pregnancy period and in general. Intervention programs designed to reduce smoking among husbands during pregnancy should focus on those with a long history of smoking, a high rate of daily cigarette consumption, and those with household registration not in Shanghai.

## INTRODUCTION

Maternal smoking during pregnancy is significantly associated with increased risks of preterm birth, low birth weight, stillbirth, and impaired fetal growth^1-3^. Although China has the world’s largest number of smokers, cigarette smoking is much less prevalent among women compared to men, 2.7% vs 52.1% in 2015, according to a large-scale national study^4^. However, exposure to environmental tobacco smoke (ETS) is quite common in China for non-smoking women during pregnancy, which can lead to similar outcomes as those of maternal smoking during pregnancy^5,6^. As reported in 2009, 69.1% of pregnant women in China were exposed to ETS at home^7^. Many non-smoking pregnant women have a high risk of exposure to ETS due to the husband’s smoking behavior, or that of other male family members who live with them. While the Chinese government recently placed a ban on smoking in public places, which might lead to some decline in smoking rates^8^, this does not affect private places such as the home, thus ETS for pregnant women often remains high^9^. Given the implementation of the ‘two-children policy’, enabling couples to have two children, the number of pregnant women is expected to increase in the future. Thus, the ability of husbands and other family members to control their smoking has extremely important implications for the pregnant wives and unborn children.

Studies in many countries have reported that the smoking rates for both pregnant women and their husbands typically decline significantly during pregnancy. A population-based mother-child cohort study in Greece completed in 2010 showed that 50% of smoking pregnant women quit smoking during the first 12 weeks of gestation^10^, while 61.9% quit in Japan^11^ and 82.9% in China^12^. Similarly, in China, 38.1% of the husbands decreased their smoking behavior during pregnancy, whilst 14.4% stopped smoking entirely during this time^13^. These studies collectively show that pregnancy is a very important period to implement health education projects targeting smokers^13^. To effectively promote smoking cessation guidance and decrease the prevalence of ETS exposure, we need to better understand those who changed behavior during pregnancy and why. Thus, the purpose of the present study was to examine the changes of smoking behavior among both pregnant women and their family members in China, and to uncover any characteristics or patterns that might be associated with these behavioral adjustments.

## METHODS

### Survey method, instrument and sample

Subjects in this study were pregnant women who visited one of nine selected institutions in Shanghai, either to set up their prenatal files, have a consultation or give birth, between July to November 2014.

A random sample of nine Women and Children’s Hospitals in Shanghai was selected. Three of these were located in downtown areas, three in suburban areas and the remainder in rural-urban fringe zones. The sample size of each institution was about 300. The total number of subjects who participated in this survey was 2831. Unfortunately, we did not collect data on those refusing to answer the questionnaire and therefore cannot provide a response rate.

This study was approved by the Medical Science Ethical committee in the authors’ organizations, and all subjects provided informed consent before participating. Each subject was asked to fill out a self-administered questionnaire while waiting at an institution for an examination. Participants were then given various infant-related products, after completing and handing in the questionnaire.

### Key measures

The questionnaire consisted of two main sections: 1) demographics and socioeconomics, and 2) smoking status. Demographic and socioeconomic questions included the pregnant woman’s age, education level, household registration, employment status, the husband’s and family’s net income per month, etc. The section on smoking status enquired about the pregnant woman’s, her husband’s and other family members’ smoking status prior to pregnancy, and their current (during pregnancy) smoking status (see supplementary file). A smoker was defined someone who smoked a cumulative total of over 100 cigarettes. Those who so indicated were asked further questions regarding their daily rate of cigarette consumption.

### Statistical analysis

Statistical analysis was performed with SPSS 20.0 software (Chicago, IL, USA). Results are reported as mean, median, or numbers with percentages. Categorical data and comparison of smoking cessation or adjustment among pregnant women, husbands, and other family members were analyzed using chi-squared analyses. A paired t-test was used to compare the rates of daily cigarette consumption during pregnancy with those prior to pregnancy. All analyses were two-tailed with a significance level of p<0.05. In multiple logistic regression, significant factors in univariate analysis were selected, and two additional factors related to smoking behavior were added into the model.

## RESULTS

### Sample characteristics

In total, 2831 eligible pregnant women completed questionnaires that were utilized in the analysis. The mean age of the women was 27.4 (SD=4.3), compared with the 29.1 (SD=4.7) average of their husbands. Out of all respondents, 994 (36.1%) were registered in Shanghai’s urban center, 179 (6.5%) in suburbs or rural areas of Shanghai, and 1581 (57.4%) were registered in other provinces but who were living in Shanghai at the time of taking the questionnaire. Corresponding to these groups, the husbands’ household registration ratio was 39.0% in the urban center, 6.1% in suburban or rural areas, and 51.0% in outlying provinces. The 25th percentile of family net income per month was 2000 RMB (low income) and the 75th percentile was 5000 RMB (medium income). A total of 648 (22.9%) of the pregnant women were unemployed during pregnancy, while only 47 (1.7%) of their husbands were unemployed. About 60.5% of the pregnant women had completed junior college education or above; for the husbands it was 66.2%. Because the sampling sites were maternity hospitals, more than half of the respondents were in their third trimester (Table 1).

**Table 1:** Demographic characteristics of respondents

	*Pregnant women N (%)*			*Husbands N (%)*		

*Characteristics*	*Total*	*Smoker*	*Non-smoker*	*Total*	*Smoker*	*Non-smoker*
Age (year)*						
<25	708 (25.2)	7 (33.3)	701 (25.2)	436 (15.8)	178 (26.3)	258 (12.3)
25-30	1478 (52.7)	11 (52.4)	1467 (52.7)	1317 (47.6)	288 (42.5)	1029 (49.2)
>30	621 (22.1)	3 (14.3)	618 (22.2)	1015 (36.7)	211 (31.2)	804 (38.5)
Miss	24 (0.8)	-	-	63 (2.2)	-	-
Household registration*						
Urban in Shanghai	994 (36.1)	4 (22.2)	990 (36.2)	1101 (40.6)	191 (28.7)	910 (44.5)
Rural in Shanghai	179 (6.5)	--	179 (6.5)	173 (6.4)	38 (5.7)	135 (6.6)
Out of Shanghai	1581 (57.4)	14 (77.8)	1581 (57.4)	1436 (53.0)	436 (65.6)	1000 (48.9)
Miss	77 (2.7)	-	-	121 (4.3)	-	-
Education *						
High school or below	1101 (39.2)	14 (66.7)	1097 (39.0)	932 (33.3)	370 (53.4)	562 (26.6)
Higher education	1708 (60.8)	7 (33.3)	1701 (61.0)	1870 (66.7)	323 (46.6)	1547 (73.4)
Miss	22 (0.8)	-	-	29 (1.0)	-	-
**Employment status**						
Employed	2162 (77.0)	18 (85.7)	2144 (76.9)	2756 (98.3)	675 (97.7)	2081 (98.5)
Unemployed	646 (23.0)	3 (14.3)	643 (23.1)	47 (1.7)	16 (2.3)	31 (1.5)
Miss	23 (0.8)	-	-	28 (1.0)	-	-
Family net income per month (RMB)*						
<5000	1494 (60.8)	15 (83.3)	1479 (60.6)	1493 (60.8)	424 (72.5)	1069 (57.2)
≥5000	963 (39.2)	3 (16.7)	969 (39.4)	962 (39.2)	161 (27.5)	801 (42.8)
Miss	374 (13.2)	-	-	376 (13.3)	-	-
Pregnancy trimester*						
First trimester	349 (12.4)	1 (4.8)	348 (12.4)	349 (12.4)	63 (9.0)	286 (13.5)
Second trimester	791 (28.0)	2 (9.5)	789 (28.1)	789 (28.0)	181 (25.9)	608 (28.6)
Third trimester	1492 (52.8)	12 (57.1)	1480 (52.8)	1492 (52.9)	387 (55.4)	1105 (52.0)
Puerperium	191 (6.8)	6 (28.6)	186 (6.8)	191 (6.8)	67 (9.6)	124 (5.8)
Miss	7 (0.2)	-	-	10 (0.4)	-	-

* p<0.05 by Chi-squared tests in husbands group.

### Smoking status before and during pregnancy

Prevalence of smoking before and during pregnancy among the pregnant women, their husbands, and other family members is given in Table 2. The smoking rate of pregnant women dropped from 1.9% (53/2831) before pregnancy to 0.7% (21/2831) during pregnancy, a significant difference (p<0.001), with a smoking cessation rate of 60.4% (32/53). Among spouses, the smoking rate dropped from 29.2% (826/2831) to 24.7% (698/2831), also a significant difference (p<0.001), with a smoking cessation rate of 15.5% (128/826). Finally, the smoking rate of the other family members who lived together with pregnant women was 20.2% (217/1073) during pregnancy and 23.8% (255/1073) before pregnancy, also a significant difference (p<0.001), with a smoking cessation rate of 14.9% (38/255).

**Table 2:** Prevalence of smoking before and during pregnancy among pregnant women, their husbands and other family members

	*Prevalence of smoking N (%) or daily cigarette consumption*		
	*Before Pregnancy*	*During Pregnancy*	*p*
Pregnant women (n=2831)	53 (1.9)	21 (0.7)	<0.001*^#^
Husbands (n=2831)	826 (29.2)	698 (24.7)	<0.001*
Other family members living together (n=1073)	255 (23.8)	217 (20.2)	<0.001*
Pregnant women’s daily cigarette consumption (n=42)	5.9±5.1	0.9±2.2	<0.001^^^
Husband’s daily cigarette consumption (n=627)	9.5±7.0	7.4±7.3	<0.001^^^
Other family members’ daily cigarette consumption (n=165)	11.4±8.5	9.5±8.7	<0.001^^^

* p values were calculated by Chi-squared tests.

# Fisher exact probability method.

^ Paired sample t-test.

Among those who continued smoking during pregnancy, the daily consumption rate of pregnant women decreased from 5.9±5.1 before pregnancy to 0.9±2.2 during pregnancy. For the husbands, the rate of daily cigarette consumption decreased from 9.5±7.0 before pregnancy to 7.4±7.3 during pregnancy. Other smoking family members’ daily cigarette consumption decreased from 11.4±8.5 before pregnancy to 9.5±8.7 during pregnancy.

### Changes in daily cigarette consumption

Table 3 shows the changes of daily cigarette consumption before and during pregnancy among pregnant women, their husbands, and other family members. Daily cigarette consumption had different proportions of reduction between the three groups of people, 85.7% for pregnant women, 46.2% for husbands, and 31.7% for other family members, respectively (p<0.001 by chi-squared test). These degrees of difference are similar to those in the group that ‘quit smoking’ entirely, 76.2% for pregnant women, 19.2% for their husbands, and 14.0% for other family members (p<0.001 by chi-squared test).

**Table 3:** Changes of daily cigarette consumption before and during pregnancy among pregnant women, their husbands and other family members

*Changes of daily cigarette consumption before and during pregnancy*	*Pregnant women (n=42 )*	*Husbands (n=627 )*	*Other family members (n=165 )*	*p (Chi-squared tests)*
Decline or quit	36 (85.7)	290 (46.2)	52 (31.5)	<0.001
Except decline	6 (14.3)	337 (53.8)	113 (68.5)	
Quit smoking	32 (76.2)	120 (19.1)	23 (13.9)	<0.001
Except quit smoking	10 (23.8)	507 (80.9)	142 (86.1)	

‘Except decline’ indicated a person whose daily cigarette consumption during pregnancy was the same or more.

### Logistic regression analysis

Table 4 shows the results of multiple logistic regression analysis for identifying factors that affect smoking rates among husbands. Findings show that the rate of smoking among husbands during pregnancy was significantly associated with household registration, cigarette consumption, and the length of time that they had previously been smoking. Compared to husbands with a relatively short history of smoking (≤5 years) or lower cigarette consumption per day (≤10 cigarettes), those with a longer history of smoking (>5 years) and high rate of daily cigarette consumption (>10 cigarettes) were more likely to continue smoking during pregnancy (OR=3.11, 95% CI: 1.82-5.31; OR=2.65, 95% CI: 1.25-5.61, respectively). Compared to husbands registered in Shanghai, husbands registered outside Shanghai were more likely to continue smoking during their wife’s pregnancy (OR=1.69, 95% CI: 1.01-2.81).

**Table 4:** Multivariable logistic regression analysis for identifying factors that affect smoking among husbands

*Variable*		*OR*	*95% CI*
**Age**	<25	1	
	25-30	0.47	0.24-0.94
	>30	0.49	0.23-1.06
**Household**	Shanghai	1	
**Registration**	Out of Shanghai	1.69	1.01-2.81
**Education level**	High school or below	1	
	Higher education	0.90	0.52-1.57
**Family net income per month (RMB)**	<5000	1	
	≥5000	1.05	0.59-1.85
**Pregnancy trimester**	First trimester	1	
	Second trimester	1.84	0.86-3.92
	Third trimester	2.10	0.99-4.46
	Puerperium	2.45	0.84-7.14
**Cigarette consumption (N/Day)**	≤10	1	
	>10	2.65	1.25-5.61
**Smoke age (years)**	≤5	1	
	>5	3.11	1.82-5.31

## DISCUSSION

This study, conducted among 2831 gestational households in Shanghai, has provided important insights into the adjustments of smoking behavior of pregnant women and their family members during the pregnancy period. As noted previously, many studies have shown that there is a significant decline in smoking rates among pregnant women during their pregnancy^14^. However, these studies are difficult to compare to China, as most have typically been carried out in high income countries and focused mainly on smoking behavior of the pregnant women themselves^15^. While very few Chinese women smoke, their exposure to ETS is quite high. ETS remains a major indoor pollutant that causes serious health problems, especially for pregnant women and unborn babies^3,16^, due to the high smoking rates of men in China^17^.

Some research has shown that the most common location for passive smoking for pregnant women was their home, mostly from their spouses and other family members^18^. Thus, this study inquired about the family unit as a whole, including pregnant women, husbands and other family members who are living together.

In this study, several unique aspects of changes in smoking behavior during pregnancy can be noted. First, the overall rates of smoking both during and before pregnancy, among all three groups of people (pregnant wives, husbands, and other family members), are comparatively low. Of the 2831 pregnant women who completed the questionnaire, only 1.9% and 0.7% stated that they smoked before and during pregnancy, respectively. The rate is lower than the results found in 2014, 0.98%^19^, and much lower than rates noted in other cities in China, such as Chongqing with 3.8% in 20159. Compared with this low rate of smoking among women, both before and during pregnancy, the smoking rate of the husbands was quite high. However, the smoking rates of the husband (24.7%) and family members who live together (20.2%) were both lower than those noted in previous studies^20^. We speculate that more and more smokers are taking into consideration the child’s health and hence are quitting smoking during this special period. Another possible reason is that health education in Shanghai might be more effective than in other cities across China.

Second, it is quite obvious from this study that the prevalence of smoking among pregnant women, husbands and other family members significantly declines during the pregnancy period. Additionally, for those that did continue smoking, their daily cigarette consumption significantly decreased, especially that of the pregnant women. Pregnancy is indeed a time when pregnant women, their husbands, and their families are all encouraged to quit smoking. But the degree of quitting between these groups was not found to be the same. Compared to each other, it was mainly the pregnant women who positively adjusted their smoking behavior during pregnancy. For husbands or family members, positive change, either quitting or reducing their rate of smoking, was much lower. Thus, it seems that attempts by health educators to reduce smoking rates during the pregnancy period should focus on the husbands and family members of the pregnant women.

Third, in the univariate analysis, the age, household registration, education level, family net income per month, and pregnancy trimester, but not employment status, were tested for association with the husband’s smoking rate during pregnancy. However, in some other studies, employment status was found to be a related factor^21,22^. We suppose the key reason for this was the comparatively small number of unemployed husbands (47, 1.7%) in this study.

Besides employment status, three other factors were found to be significant and worthy of discussion. The first of these was the location of household registration. Compared with husbands registered not in Shanghai, those registered in Shanghai were less likely to be smokers. We suppose this is perhaps due to the relatively good health promotion activities that are carried out for local people in Shanghai, compared with other programs, or lack thereof, in other cities in China.

Additionally, education level was found to be significant. Husbands with a lower level of education (high school or below) were more likely to be smokers compared to those with a higher level of education. A similar result was found regarding the third significant factor, family net income per month, the lower the net income the more likely a husband was to be a smoker. This is perhaps due to the commonly observed positive correlation between higher education and higher income. Husbands with higher education most likely have more knowledge, or at least more access to knowledge, about the harms of smoking. However, many studies have shown that there exists much controversy about the influence of income and education on smoking, due to different results with different samples^23-25^.

Considering the possible association between these factors, we conducted a multiple regression analysis, including the factors that were statistically significant in the univariate analysis, and two other important factors related to smoking behavior (smoking age and rate of cigarette consumption). These results confirmed that husbands whose household was registered in Shanghai were less likely to be smokers. In addition, besides the location of their household registration, only the length of time of their smoking behavior and their rate of cigarette consumption were found to be influencing factors. We are of the opinion that both the length of smoking history and the rate of daily cigarette consumption likely reflect the degree of nicotine addiction of the respondent^26^. Consequently, husbands with a high level of daily cigarette consumption (>10 cigarettes/day) were less likely to quit smoking during pregnancy, which is in line with a similar study conducted in England^22^.

This study has some limitations. First, the present study was a cross-sectional study that investigated only the prevalence of smoking during pregnancy, but did not include a follow-up study after pregnancy. Second, the use of a questionnaire could have led to memory bias; some respondents may not have answered the questions truthfully or precisely, particularly regarding family economic circumstances or daily cigarette consumption. Also, the smoking status of husbands and other family members were reported by the pregnant women and not asked directly to the individuals. Third, our study is not nationally representative; the sample consisted exclusively of pregnant women and their families who were visiting one of the selected nine Women and Children’s Hospitals in Shanghai. Other regions and cities in China may well have different levels of smoking, and also different adjustments to pregnancy. Fourth, due to the low smoking rate among the women surveyed, the sample of pregnant women who continued smoking was too small to carry out univariate analysis, as was completed for the husbands. Fifth, some other factors, such as pregnant women’s knowledge and attitude toward ETS, were not measured in the current study.

## CONCLUSIONS

Our study shows that in the Shanghai region, the prevalence of smoking among pregnant women, their husbands and other family members significantly declines during pregnancy. Thus, pregnancy is a key period in which to conduct health education regarding how to control smoking for families with pregnant women. In addition, intervention programs designed to counteract the husband’s smoking during pregnancy should focus on those husbands with a long history of smoking, a high rate of daily cigarette consumption, and whose household registration is not in Shanghai.

## References

[1] Agrawal A, Scherrer JF, Grant JD, Sartor CE, Pergadia ML, Duncan AE (2010). The effects of maternal smoking during pregnancy on offspring outcomes. Prev Med.

[2] Chelchowska M, Ambroszkiewicz J, Mazur J, Lewandowski L, Maciejewski TM, Ołtarzewski M (2014). Effect of tobacco smoking on the maternal and fetal adipokine axis in relation to newborn birth weight and length. Przegl Lek.

[3] Bjornholt SM, Leite M, Albieri V, Kjaer SK, Jensen A (2016). Maternal smoking during pregnancy and risk of stillbirth: results from a nationwide Danish register-based cohort study. Acta Obstet Gynecol Scand.

[4] World Health Organization WHO report on the global tobacco epidemic 2017.

[5] Been JV, Mackenbach JP, Millett C, Basu S, Sheikh A (2015). Tobacco control policies and perinatal and child health: a systematic review and meta-analysis protocol. BMJ Open.

[6] Ion RC, Wills AK, Bernal AL (2015). Environmental Tobacco Smoke Exposure in Pregnancy is Associated With Earlier Delivery and Reduced Birth Weight. Reproductive Sciences.

[7] Yao T, Lee AH, Mao Z (2009). Potential unintended consequences of smoke-free policies in public places on pregnant women in China. Am J Prev Med.

[8] Qian XL, Gu HY, Wang L, Fu CW, Wang X (2017). Influence of The Regulations of Shanghai Municipality on Smoking Control in Public Places on residents’ smoking behavior. Journal of Environmental & Occupational Medicine.

[9] Xu X, Rao Y, Abdullah AS, Sharma M, Guo JJ, Zhao Y (2016). Preventive behaviours in avoiding indoor secondhand smoke exposure among pregnant women in China. Tobacco Control.

[10] Vardavas CI, Chatzi L, Patelarou E, Plana E, Sarri K, Kafatos A (2010). Smoking and smoking cessation during early pregnancy and its effect on adverse pregnancy outcomes and fetal growth. Eur J Pediatr.

[11] Kaneita Y, Tomofumi S, Takemura S, Suzuki K, Yokoyama E, Miyake T (2007). Prevalence of smoking and associated factors among pregnant women in Japan. Prev Med.

[12] Xu X, Rao Y, Wang L, Liu S, Guo JJ, Sharma M (2017). Smoking in pregnancy: a cross-sectional study in China. Tob Induc Dis.

[13] Fu C, Chen Y, Wang T, Edwards N, Xu B (2008). Exposure to environmental tobacco smoke in Chinese new mothers decreased during pregnancy. J Clin Epidemiol.

[14] Lauria L, Lamberti A, Grandolfo M (2012). Smoking Behaviour before, during, and after Pregnancy: The Effect of Breastfeeding. The Scientific World Journal.

[15] Dherani M, Zehra SN, Jackson C, Satyanaryana V, Huque R, Chandra P (2017). Behaviour change interventions to reduce second-hand smoke exposure at home in pregnant women - a systematic review and intervention appraisal. Bmc Pregnancy & Childbirth.

[16] Huang J, Wen G, Yang W, Yao Z, Wu C, Ye X (2017). The association between second-hand smoke exposure and depressive symptoms among pregnant women. Psychiatry Res.

[17] Wang Z, Koenig HG, Al Shohaib S (2015). Religious involvement and tobacco use in mainland China: a preliminary study. BMC Public Health.

[18] Yang L, Tong EK, Mao Z, Hu TW (2010). Exposure to secondhand smoke and associated factors among non-smoking pregnant women with smoking husbands in Sichuan province, China. Acta Obstetricia Et Gynecologica Scandinavica.

[19] Chen H, Chen XW, Shi LL, Cai YY (2015). Analysis of smoking status and influencing factors of families with pregnant women in Shanghai. Journal of Shanghai Jiaotong University.

[20] Li Z, Yao Y, Yu Y, Shi J, Liu Y, Tao Y (2015). Prevalence and Associated Factors of Passive Smoking among Women in Jilin Province, China: A Cross-Sectional Study. International Journal of Environmental Research & Public Health.

[21] Ham DC, Przybeck T, Strickland JR, Luke DA, Bierut LJ, Evanoff BA (2011). Occupation and workplace policies predict smoking behaviors: analysis of national data from the current population survey. Journal of Occupational & Environmental Medicine.

[22] Syamlal G, Mazurek JM, Hendricks SA, Jamal A (2015). Cigarette Smoking Trends Among U.S. Working Adult by Industry and Occupation: Findings From the 2004-2012 National Health Interview Survey. Nicotine & Tobacco Research.

[23] Tenn S, Herman DA, Wendling B (2010). The role of education in the production of health: an empirical analysis of smoking behavior. Journal of Health Economics.

[24] Ergin I, Hassoy H, Tanik FA, Aslan G (2010). Maternal age, education level and migration: socioeconomic determinants for smoking during pregnancy in a field study from Turkey. BMC Public Health.

[25] Blakely T, van der Deen FS, Woodward A, Kawachi I, Carter K (2013). Do changes in income, deprivation, labour force status and family status influence smoking behaviour over the short run? Panel study of 15,000 adults. Tobacco Control.

[26] Hu MC, Davies M, Kandel DB (2011). Epidemiology and Correlates of Daily Smoking and Nicotine Dependence Among Young Adults in the United States. American Journal of Public Health.

